# A Rapid, Physiologic Protocol for Testing Transcriptional Effects of Thyroid-Disrupting Agents in Premetamorphic *Xenopus* Tadpoles

**DOI:** 10.1289/ehp.7992

**Published:** 2005-07-11

**Authors:** Nathalie Turque, Karima Palmier, Sébastien Le Mével, Caroline Alliot, Barbara A. Demeneix

**Affiliations:** Unité Mixte de Recherche, Centre National de la Recherche Scientifique, Evolution des Régulations Endocriniennes, Department of Regulations, Development and Molecular Diversity, Museum National d’Histoire Naturelle, Paris, France

**Keywords:** acetochlor, endocrine disruption, germinal transgenesis, green fluorescent protein, metamorphosis, somatic gene transfer, thyroid, transcription, *Xenopus laevis*

## Abstract

Increasing numbers of substances present in the environment are postulated to have endocrine-disrupting effects on vertebrate populations. However, data on disruption of thyroid signaling are fragmentary, particularly at the molecular level. Thyroid hormone (TH; triiodothyronine, T_3_) acts principally by modulating transcription from target genes; thus, thyroid signaling is particularly amenable to analysis with a transcriptional assay. Also, T_3_ orchestrates amphibian metamorphosis, thereby providing an exceptional model for identifying thyroid-disrupting chemicals. We combined these two advantages to develop a method for following and quantifying the transcriptional action of T_3_ in *Xenopus laevis* tadpoles. This technology provides a means of assessing thyroid activity at the molecular level in a physiologically relevant situation. Moreover, translucent tadpoles are amenable to “on-line” imaging with fluorescent reporter constructs that facilitate *in vivo* measurement of transcriptional activity. We adapted transgenesis with TH-responsive elements coupled to either luciferase or green fluorescent protein to follow T_3_-dependent transcription *in vivo*. To reduce time of exposure and to synchronize responses, we optimized a physiologic pre-treatment protocol that induced competence to respond to T_3_ and thus to assess T_3_ effects and T_3_ disruption within 48 hr. This pretreatment protocol was based on a short (24 hr), weak (10^−12^ M) pulse of T_3_ that induced TH receptors, facilitating and synchronizing the transcriptional responses. This protocol was successfully applied to somatic and germinal transgenesis with both reporter systems. Finally, we show that the transcriptional assay allows detection of the thyroid-disrupting activity of environmentally relevant concentrations (10^−8^ M) of acetochlor, a persistent herbicide.

The thyroid hormone (TH) triiodothyronine (T_3_) is critical to vertebrate development and growth, playing vital roles during central nervous system (CNS) development and during organogenesis of heart, muscles, bones, and lungs. In both developing and mature organisms, numerous physiologic functions are regulated by T_3_ availability, including energy metabolism, thermogenesis, pituitary hormone production, and lipogenesis (Yen 2001). The most striking example of T_3_ action in vertebrates is anuran amphibian metamorphosis, one of the best-studied hormone-regulated developmental processes. Amphibian metamorphosis is totally dependent on T_3_ and is associated with dramatic morphologic and physiologic changes, including cell death, division, or differentiation (Dodd and Dodd 1976).

The total dependence of amphibian metamorphosis on TH has logically led to the suggestion that metamorphosis can be used to assess TH disruption. Accordingly, the *Xenopus* embryonic metamorphosis assay (XEMA) has been proposed by the Organization for Economic Cooperation and Development (OECD) Task Force on Endocrine Disrupters Testing and Assessment as an *in vivo* assay for identification of substances with potential to disrupt functions of the thyroid system. In this *in vivo* test, several morphologic and histologic parameters are used to define the potential of a chemical to perturb the thyroid axis (OECD 2004). Because the test covers most of the natural metamorphic process, it takes at least 4 weeks. In contrast, transcriptional responses to T_3_ are much more rapid, with changes being measurable within hours or days. Indeed, T_3_ actions are mainly mediated by their nuclear receptors (TH receptors, TRs), ligand-dependent transcription factors. In vertebrates, two genes encode TRs: *TR-*α and *TR-*β (Mangelsdorf et al. 1995). Generally, TRs form heterodimers with the 9-*cis*-retinoic acid receptor and interact with comodulator complexes, thereby repressing or activating transcription. Heterodimers bind to TH response elements (or T_3_ responsive element; TRE) in target genes.

We chose to take advantage of the speed of the transcriptional responses to TH and the ease of measuring reporter gene activities, such as luciferase (luc) and green fluorescent protein (GFP), using transgenic approaches. Moreover, both somatic (de Luze et al. 1993; Nakajima and Yaoita 2003; Ulisse et al. 1996) and germinal transgenesis (Coen et al. 2001; Huang and Brown 2000; Marsh-Armstrong et al. 1999; Oofusa et al. 2001) are now widely applied to *Xenopus laevis* for dissecting TH-dependent regulations during metamorphosis. The transgenic models we optimized here are based on the fundamental design of composite reporter gene constructs with a hormone-sensitive regulatory region upstream of a fluorescent protein cDNA. We started from the premise that following transcriptional responses *in vivo* is often marred by high variability. In the case of following TH responses in tadpoles, this variability could be due to variations in endogenous TR levels. Because TR-β is strongly inducible by T_3_ itself, we chose to prime tadpoles to respond to an ulterior T_3_ exposure with a short, weak pulse of T_3_ that was then fully rinsed out. This protocol produced rapid (48 hr), robust, and reproducible responses to TH agonists. Applying this protocol to germinally transgenic tadpoles, we were able to reveal the actions of the preemergent herbicide acetochlor through increased TH responses.

## Materials and Methods

### Plasmid constructs.

The –246 to +130 bp sequence of the TH/bZIP promoter (GenBank accession no. U37375; GenBank 2004) was amplified by polymerase chain reaction (PCR) from *X. laevis* genomic DNA using the primers 5′-CTGTTATATAGAGGCAGAGGG-3′ and 5′-CTATACCTGAATGGGCAGCAG-3′, and then cloned into pGEMt-easy vector (Promega, Lyon, France). A *Sac*II-*Pst*I–digested fragment was excised and cloned into pBluescript (Promega). A *Sac*I-*Hin*dIII–digested fragment was excised and cloned into pGL2 basic vector (Promega), producing TH/bZIP-luc.

To obtain the TH/bZIP-eGFP (enhanced GFP) transgene, we proceeded in two steps. We cloned a *Sal*I-*Apa*I eGFP cDNA and the SV40 polyA signal fragment into the TH/bZIP promoter containing pBluescript. A large *Sac*I-*Apa*I fragment was excised and cloned into a pBluescript vector with two insulators. To this end, a 1,668-bp fragment of the lysozyme gene (GenBank accession no. X98408) from chicken genomic DNA (Stief et al. 1989) was amplified by PCR using the primer 5′-TGACTCGAGGGATCCATAATATAACTGTACC-3′ and 5′-TGAGGTACCAAGCTTAAAAGATTGAAGCAC-3′. One insulator copy was cloned into *Xho*I and *Kpn*I sites of the pBluescript vector, and the second was cloned into the *Sma*I site. The complete vector with the two copies of insulators was linearized with EcoRV, and the large *Sac*I-*Apa*I fragment corresponding to the eGFP cDNA and SV40 polyA signal was inserted.

The γ-crystallin promoter coupled to a RedFP (red fluorescent protein) plasmid was a gift from L. Zimmermann (Medical Research Council, London).

### Animals and treatments.

We obtained sexually mature *X. laevis* frogs from d’Elevage de Xénope du Centre National de la Recherche Scientifique (Montpellier, France). Tadpoles were raised in dechlorinated and deiodinated tap water (1:2) and fed with nettle powder (Vallée, Chanzeaux, France). Tadpoles were staged according to Nieuwkoop and Faber (1956; NF staging). The care and treatment of animals used in this study were in accordance with institutional and national guidelines (Sciences et Médecine des Animaux de laboratoire à l'ENVL 2005).

T_3_, 3,5,3′-triiodothyroacetic acid (TRIAC), and acetochlor were purchased from Sigma (St. Quentin Fallavier, France).

### Somatic gene transfer and germinal trans-genesis.

Somatic gene transfer in *Xenopus* muscle and brain was performed as described previously (de Luze et al. 1993; Ouatas et al. 1998; Trudeau et al. 2004).

Germinally transgenic tadpoles were produced by restriction enzyme-mediated integration nuclear transplantation according to Kroll and Amaya (1996), with the following modifications: sperm was purified by centrifugation on a two-layer discontinuous Percoll (Sigma) gradient before the permeabilization step, which was performed with digitonin (Sigma) instead of lysolecithin. Two plasmids were used: TH/bZIP-eGFP plasmid and a γ-crystallin promoter coupled to a RedFP plasmid, which is expressed only in the eye. This latter plasmid allows selection of transgenic F_0_ tadpoles during early development before the TH/bZIP driven green fluorescence appears in the tadpole body.

### Imaging.

Images were captured using an Olympus fluorescent dissecting microscope equipped with an Olympus video camera DP50 (Olympus, Rungis, France). Before photographing, germinally transgenic tadpoles NF stage 52 were anaesthetized in 0.1% tricaine methanesulfonate (MS-222; Sigma) and the skull opened to expose the brain. All pictures were taken with the same parameters (32 × objective and 5-sec exposure time). Quantification was performed using ImageJ software (Rasband 1997). Data are expressed in relative units of fluorescence.

### Luciferase activity.

Tadpoles were sacrificed by decapitation after anesthesia in 0.1% MS-222. Tissues were dissected, frozen in liquid nitrogen, and stored at –80°C until assayed according to the manufacturer’s instructions (Promega) as previously reported (de Luze et al. 1993). Luciferase activity is expressed as relative light units (RLU). Because in some experiments tadpoles vary in size, luciferase values were normalized against protein content. Protein was measured according to the manufacturer’s instructions (BioRad, Marnes-La-Coquette, France).

### Statistical analysis of results.

*In vivo* gene transfer results are expressed as mean ± SE per group. Differences between means were analyzed by Student’s *t*-test or analysis of variance and the Tukey-Kramer test where appropriate. Differences were considered significant at *p* < 0.05. In many cases, typical experiments are shown, each experiment having been repeated at least twice (with *n* ≥ 8 tadpoles/experiment) and providing the same results.

### RNA extraction and semiquantitative reverse transcriptase (RT)-PCR analysis.

Tadpole tails were harvested into RNAlater (Ambion, Huntingdon, United Kingdom) at 4°C. Total RNA was extracted using RNAble reagent (Eurobio, Les Ulis, France) following the manufacturer’s protocol. Reverse transcription was performed on 2 μg RNA, in 20 μL final volume. Primer hybridization on RNAs was done by mixing total RNAs with specific reverse primers (2 μM each): for TR-β, 5′-CTTTTCTATTCTCTCCACGCTAGC-3′; for the internal control Rpl8, 5′-GACGACCAGTACGACGA-3′ (Havis et al. 2003). Mixes were incubated (2 min, 65°C) and then cooled to room temperature. A 10-μL mix containing 4 μL reverse transcription buffer (Invitrogen, Cergy Pontoise, France), 1 μL dNTP (dATP, dTTP, dCTP, and dGTP, 25 mM each; Pharmacia, Saclay, France), 2 μL 0.1 M dithiothreitol (Invitrogen), and 0.5 μL reverse transcriptase (RT) SuperScript II (5 U/μL; Invitrogen) was added to hybrid primers/RNA before incubation (1 hr, 45°C). After reverse transcription, we used 2 μL of each cDNA sample and 0.1 μL (0.1 μCi) of [α-^32^P] dCTP for PCR in a final volume of 50 μL containing 25 μL PCR Master Mix (Abgene, Courtaboeuf, France), 2 μL of each primer (forward and reverse for the gene of interest and the internal control at 2 μM each; TR-β forward, 5′-ATAGTTAATGCGCCCGAGGGTGGA-3′; and the internal control, Rpl8 forward, 5′-AAAGAGAAACTGCTGGC-3′). The PCR reaction consisted of 22 cycles of 60 sec at 94°C, 1 min at 55°C, and 1 min at 72°C (Robocycler; Stratagene, Amsterdam, The Netherlands). Fifteen microliters of PCR products was resolved in 6% acrylamide-trisborate-EDTA buffer gels and autoradiographed.

## Results

### Treatment with a rapid, weak pulse of T_3_ induces competence to respond and synchronizes T_3_ transcriptional responses in somatic gene transfer.

**Figure 1:**
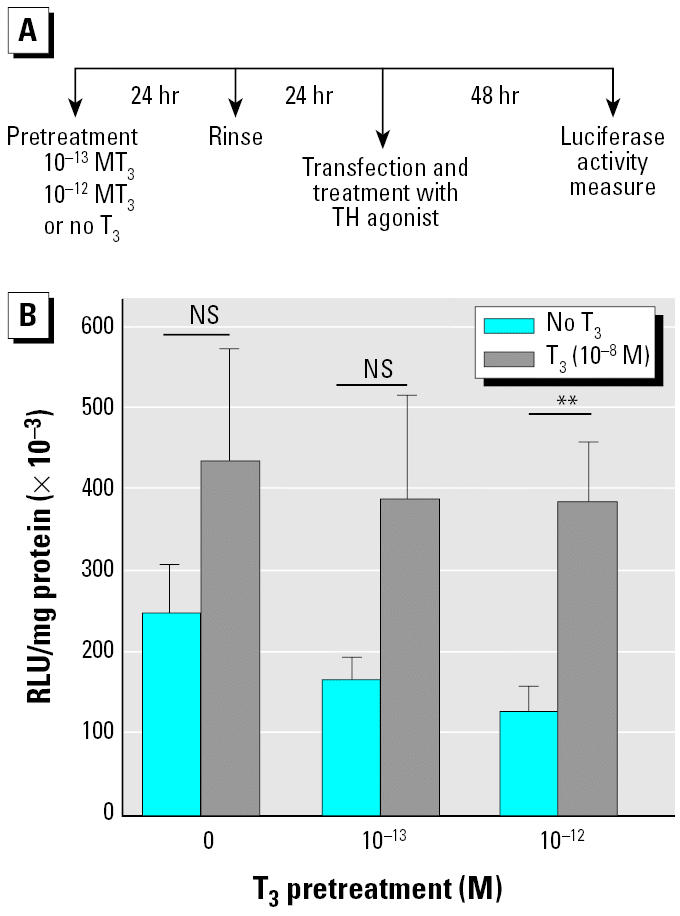
A significant transcriptional response induced by a 24-hr pretreatment, or priming, pulse of 10^−12^ M T_3_ within 48 hr in tadpoles later exposed to 10^−8^ M T_3_. NS, not significant. (*A*) A linear schema indicating the timing of the pretreatment/rinse/exposure protocol. Tadpoles were pretreated with or without T_3_ 24 hr, rinsed in water, and fed during 24 hr before injection with 200 ng TH/bZIP-luc construct in the caudal muscle. (*B*) Measured TH/bZIP-luc transcription in pretreated tadpoles exposed or not exposed to T_3_ (10^−8^ M) for 48 hr. Values shown are mean ± SE (*n* = 12/group). In each case, the experiment was repeated three times, providing similar results. **p < 0.01.

Our overall aim in these experiments was to exploit the T_3_-dependent inducibility of the *TR-*β gene to prime tadpoles so as to have access to a model that could reveal rapid, robust, and reproducible TH transcriptional responses. This priming, or pretreatment procedure, was considered a prerequisite to using premetamorphic (NF stage 54) tadpoles in a reporter gene assay, because intragroup variability can be quite high (de Luze et al. 1993). Using the promoter of the *TH/bZIP* gene, which is a T_3_ target gene encoding a transcription factor (Furlow and Brown 1999), we compared the effects of a short pulse (24 hr) of 10^−13^ M or 10^−12^ M T_3_ for the pre-treatment against unpretreated, control tadpoles. After a 24-hr rinse period, both sets of tadpoles (pretreated and control) were used for somatic gene transfer in the caudal muscle and exposed to T_3_ (10^−8^ M) for 2 days. We found 2-fold increases in mean levels of TH/bZIP expression in caudal muscle of control tadpoles and those given a pretreatment pulse of T_3_ at 10^−13^ M (Figure 1B). However, these differences were not significant (*p* > 0.05). In contrast, in tadpoles transiently pretreated with 10^−12^ M T_3_, 2 days of exposure to 10^−8^ M T_3_ induced a nearly 4-fold increase in the transcriptional response of the TH/bZIP-luc construct. This increase was very significant (*p* < 0.01). Interestingly, the basal levels of transcription from the *TH/bZIP* promoter decreased as a function of pretreatment pulse concentration, most probably reflecting stronger repression of basal expression by the unliganded TR (Sachs 2004).

### A weak pulse of T_3_ induces TR-β expression in caudal muscle of tadpoles within 6 hr.

**Figure 2:**
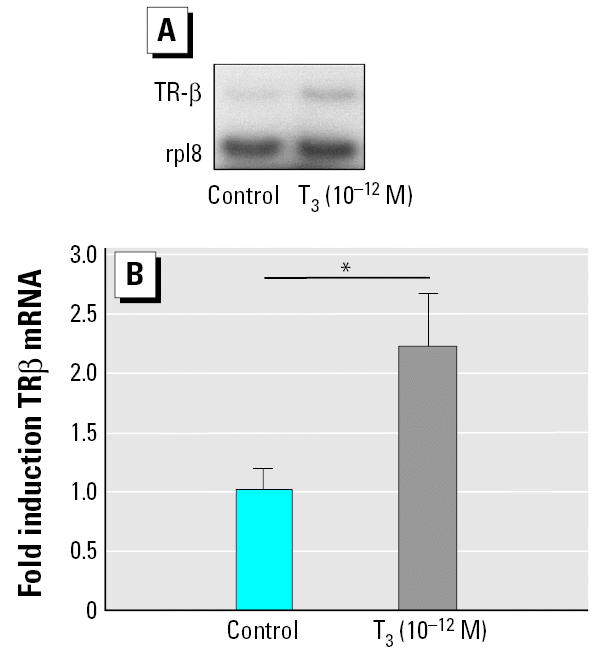
Induction of TR-β expression by a weak T_3_ pulse. To evaluate the effect of the pretreatment protocol on TR-β expression, tadpoles were exposed for 6 hr to 10^−12^ M T_3_. Total RNA was extracted from caudal muscles and used for RT-PCR analysis of TR-β expression. Rpl8 was used as the internal control. (*A*) Typical scan obtained after 22 cycles of PCR amplification. (*B*) Same results quantified by Phosphoimager scanning (Molecular Dynamics, Sunnyvale, CA, USA). Values shown are mean ± SE of five independent experiments expressed as multiples of induction, where 1 is equal to expression in the absence of T_3_ (untreated tadpole; Rpl8) as the control level. For each sample, densitometry readings were normalized against the value for Rpl8. *p < 0.05.

To verify that the pretreatment protocol was indeed inducing TR-β, we followed TR expression in caudal muscle of pretreated tadpoles using semiquantitative RT-PCR. Figure 2 shows that there is a significant, 2.2-fold induction in TR-β expression within 6 hr of exposure of NF stage 54 tadpoles to 10^−12^ M T_3_. Given this finding, and the robust response produced by pretreatment, all the following experiments were performed on animals pretreated with 10^−12^ M T_3_ during 24 hr, followed with a rinse of 24 hr before exposure to TH agonists for 48 hr.

### The somatic gene transfer method allows dose-dependent detection of TH agonists in brain and muscle.

**Figure 3:**
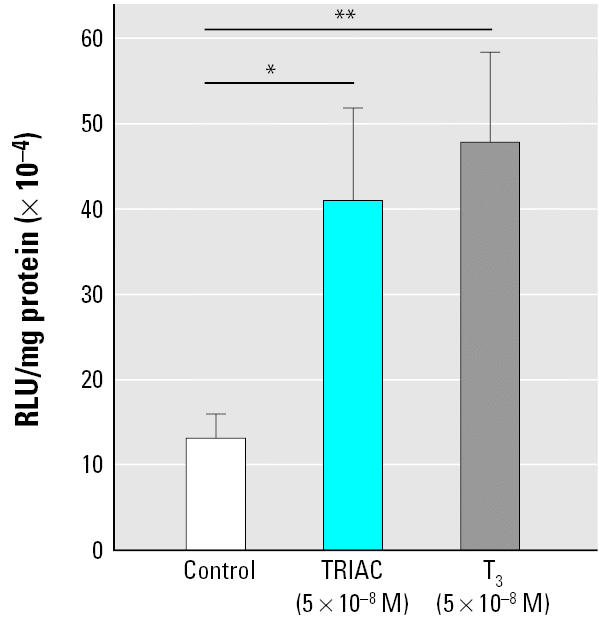
Action of TH agonists assessed using the somatic gene transfer method and pretreatment. Tadpoles were pretreated 24 hr with 10^−12^ M T_3_ and then rinsed and fed during 24 hr before injection of 200 ng TH/bZIP-luc construct in the caudal skeletal muscle. TH/bZIP-luc transcription was measured in injected tadpoles exposed to 5 × 10^−8^ M T_3_ or 5 × 10^−8^ M TRIAC for 48 hr. Values shown are mean ± SE (*n* = 12/group). Each experiment was repeated three times, providing similar results. *p < 0.05. ** < 0.01.

We used somatic gene transfer with the pretreatment protocol to test transcriptional responses to other TH agonists. Figure 3 shows that pretreated tadpoles exposed to TRIAC (5 × 10^−8^ M) for 48 hr displayed a 3-fold increase in transcription from the *TH/bZIP* promoter (*p* < 0.05) compared with controls. Similar results were found with 3,5,3′,5′-tetraiodothyronine (T_4_; data not shown).

**Figure 4:**
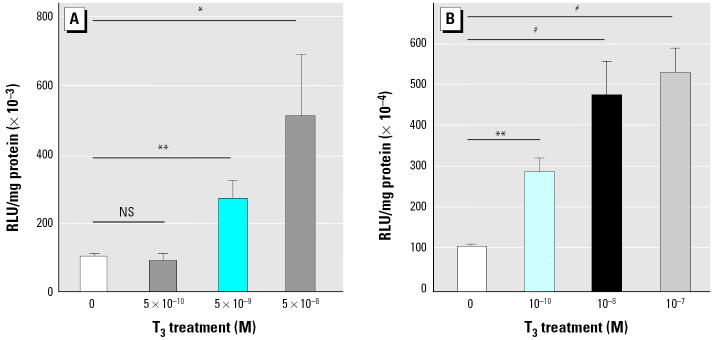
Dose dependency of TH effects on transcriptional responses. NS, not significant. (*A*) Transcriptional response in tadpoles pretreated for 24 hr with 10^−12^ M T_3_ and then rinsed and fed during 24 hr before injection of 200 ng TH/bZIP-luc construct in the caudal skeletal muscle. The TH/bZIP-luc transcription was measured in injected tadpoles exposed or not exposed to 5 × 10^−8^ M, 5 × 10^−9^ M, or 5 × 10^−10^ M T_3_ for 48 hr. (*B*) Transcriptional response in tadpoles injected with 500 ng TH/bZIP-luc construct in the brain, pretreated 24 hr with 10^−12^ M T_3_, and then rinsed and fed during 24 hr before exposure to 10^−7^ M, 10^−8^ M, or 10^−10^ M T_3_. TH/bZIP-luc transcription was measured after 48 hr. Values shown are mean ± SE (*n* = 12/group). Each experiment was repeated three times, providing similar results. *p < 0.05. **p < 0.01. ^#^*p* < 0.001.

In order to test the sensitivity of the somatic gene transfer method and the eventual tissue specificity in transcriptional response to TH agonists, we compared responses in caudal muscle or the brain of pretreated tadpoles. As shown in Figure 4A, in caudal muscle, a 5 × 10^−10^ M T_3_ exposure did not significantly increase transcription, but 5 × 10^−9^ M and 5 × 10^−8^ M T_3_ induced significant responses of 3-fold (*p* < 0.01) and 5-fold (*p* < 0.05), respectively. Figure 4B shows results from similar experiments performed using somatic gene transfer in the tadpole brain. All the T_3_ concentrations used (10^−10^ M, 10^−8^ M, and 10^−7^ M) gave very significant transcriptional responses from the TH/bZIP-luc construct: 3-fold (*p* < 0.01), 5-fold (*p* < 0.001), and 5.5-fold (*p* < 0.001), respectively. Thus, the brain is a more sensitive TH target than is muscle, in terms of transcriptional responses from the TH/bZIP-luc construct.

### The pretreatment protocol can be used on germinal transgenic tadpoles.

Because the TH/bZIP construct used in somatic gene transfer experiments had proven suitable for detecting of T_3_ agonists in muscle and brain, we used the same construct in germinal trans-genesis (Kroll and Amaya 1996). We prepared a vector with the TH/bZIP-GFP chimeric gene between two copies of the lysozyme chicken gene as insulators (Stief et al. 1989). As a means of verifying transgenesis, we injected a second plasmid containing the γ-crystallin promoter coupled to a RedFP. This plasmid is expressed early in the eye and allows selection of transgenic F_0_ tadpoles before the TH/bZIP-dependent green fluorescence appears in the tadpole body.

**Figure 5:**
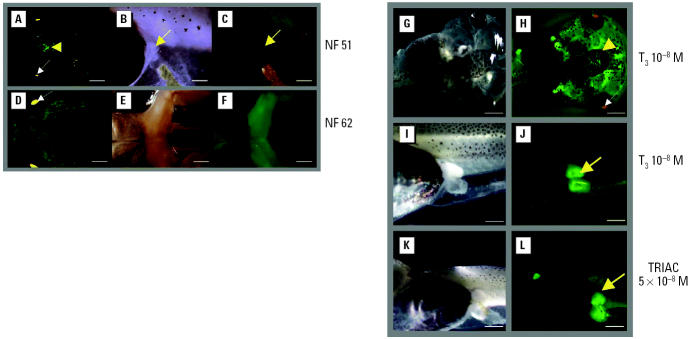
(*A–F*) Transcription responses of the TRE-containing transgene to endogenous TH induced by natural metamorphosis in germinally transgenic F_0_ embryos at NF stages 51 (*A–C*) and 62 (*D–F*). (*A, D*) Brain. (*B–F*) Limb buds. TH/bZIP-eGFP is expressed first in the brain (*A*, yellow arrowhead) and then in other tissues, and persists throughout larval development (*A, D*). No fluorescence above background is present in limb buds at NF stage 51 (*C*, yellow arrow). The signal increases throughout larval development until metamorphosis is reached (NF stage 62), when it increases strongly (*F*). Bars = 1.6 mm (*A*); 0.5 mm (*B, C*); 1.8 mm (*D*); 1.7 mm (*E, F*). (*G–L*) The pretreatment protocol significantly reduced time for response to T_3_ and to TH analogues in TH/bZIP-eGFP transgenic F_0_ tadpoles (*G, H*, head; *I–L*, hindlimb). Tadpoles were pretreated for 24 hr with 10^−12^ M T_3_ at NF stages 51–52, and then rinsed and fed during 24 hr before being exposed to 10^−8^ M T_3_ (*J*) or to 5 × 10^−8^ M TRIAC (*L*). Fluorescence in the CNS (*H*) and in hindlimb buds (*J, L*) was observed after 2 days of treatment. Yellow arrows indicate limb buds; arrowhead indicates the brain area. White arrows indicate crystallin-RFP expression in the eye. Bars = 2 mm (*G, H*); 0.8 mm (*I–L*).

We first followed TH/bZIP-driven fluorescence during early development and metamorphosis, focusing on the brain and on limb buds. In limb buds, the transgene is barely expressed at NF stage 51, and expression remains weak until NF stages 61–62. During metamorphosis, the signal intensifies. The transgene is expressed in the CNS at NF stage 51, and the fluorescent signal increases gradually through each metamorphic stage (Figure 5A). However, TH/bZIP expression remains at low enough levels in the brain so as not to interfere with fluorescence induction by TH agonists.

A number of F_0_ germinally transgenic tadpoles were selected and bred to obtain F_1_ tadpoles. We next exposed F_1_ transgenic tadpoles to T_3_ (10^−8^ M) using the pretreatment protocol. We confirmed that a short (24 hr) pretreatment with a low concentration of T_3_ (10^−12^ M), followed by a rinse, permits a rapidly detectable fluorescent signal in limb buds (forelimb and hindlimb buds) (Figure 5B), that is, after 48-hr exposure to T_3_ (10^−8^ M). In contrast, if the pretreatment protocol is not used, 4 days of exposure to T_3_ (10^−8^ M) is required to obtain a significant induction (data not shown). Fluorescence was induced in the brain, the olfactory nerves, and the gills (Figure 5B). TRIAC (5 × 10^−8^ M) induced similar responses in the brain (data not shown) and limb buds (Figure 5B).

### Acetochlor disruption of thyroid signaling can be assessed within 48 hr.

**Figure 6:**
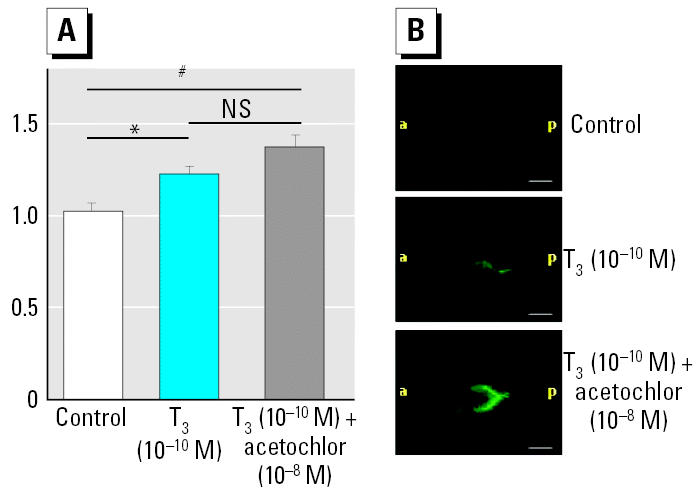
The TH enhancing activity of the pesticide acetochlor revealed in the brains of germinally transgenic premetamorphic tadpoles. Abbreviations: a, anterior; NS, not significant; p, posterior. (*A*) NF stage 50–52 germinally transgenic tadpoles bearing a TH/bZIP-eGFP transgene were pretreated for 24 hr with 10^−12^ M T_3_ and then rinsed and fed for 24 hr.Fluorescence was measured in tadpole brains after 48 hr exposure to 10^−10^ M T_3_ or to 10^−10^ M T_3_ plus 10^−8^ M acetochlor. Values shown are mean ± SE of three experiments expressed as multiples of induction, where 1 = control expression in the absence of T_3_. Data were normalized and analyzed by Student’s *t*-test. (*B*) Representative photographs of strongly fluorescent tadpoles brain from each group (*n* = 15 tadpoles per group). Bars = 0.4 mm. *p < 0.05. ^#^*p* < 0.001.

Because the pre-treatment protocol can be used with germinally transgenic tadpoles to reveal T_3_ effects, we applied it to NF stage 52 tadpoles to assess the potential thyroid-disrupting effects of the herbicide acetochlor. Pretreated tadpoles were exposed to 10^−10^ M T_3_ or to 10–10 M T_3_ plus 10^−8^ M acetochlor for 48 hr. As shown in Figure 6A, the 20% increase in fluorescence in brains of germinally transgenic tadpoles was amplified by addition of 10^−8^ M acetochlor (*p* < 0.001 vs. controls). Figure 6B shows examples of germinally transgenic tadpoles brains that were quantified using ImageJ software.

## Discussion

A central part of this study included establishing a rapid and sensitive method for assaying TH agonist activity within a shorter time frame than the several weeks needed to record TH effects on morphologic changes. This objective required fulfillment of numerous criteria: low background with no interference from endogenous hormone, robust and statistically significant responses, dose dependence, and low threshold. To avoid interference from endogenous hormone, we used euthyroid tadpoles at stages of development where TH levels are naturally low (Leloup and Buscaglia 1977). We had previously found that, when using euthyroid tadpoles in the somatic gene transfer test, a minimum of 4 days was necessary for significant induction of T_3_-dependent transcription (de Luze et al. 1993). Moreover, variability of responses among individuals was high. We theorized that the delay to response and the range of response levels could be due to lack of competence to respond to TH, possibly due to insufficient TRs in the target tissue, caudal muscle. Indeed, many experimental data have shown that TRs are expressed only at low levels before premetamorphosis, after which TR-β is strongly induced by the T_3_ signal, the *TR*-β genes in *X. laevis* having complex promoters containing multiple positive TREs (Urnov and Wolffe 2001). To overcome this TR insufficiency, we chose to treat tadpoles with a brief, weak pulse of T_3_ to induce competence to respond to a later exposure to TH. The logic was that the short pulse of T_3_ should synchronize and harmonize the tadpole responses by up-regulating expression of TR-β, and possibly cofactors, thus facilitating TH responses. Further, given that the animals were then rinsed, it was expected that any hormone taken up would be degraded during the 24-hr rinse period, given that the half-life of T_3_ in most vertebrates is around 18–24 hr (Van Middlesworth 1974).

We established that a short priming or pre-treatment (24 hr) used with a weak concentration of T_3_ (10^−12^ M) induced an up-regulation of TR-β expression and synchronized responses. These low concentrations and short exposure times do not induce any major morphologic changes in the tadpoles. TR-β transcript levels were increased 2-fold within 6 hr by 10^−12^ M T_3_ (Figure 2). These data confirmed previous experiments on whole tail tissue (Havis et al. 2003) or on tail tissues undergoing apoptosis (Helbing et al. 2003). In both cases, the authors observed a significant increase of TR-β expression, however, with much higher T_3_ concentrations (10^−8^ M T_3_ and 10^−7^ M T_3_) than we used.

We next determined whether responses were physiologic. We show that this is the case, in that they are dose dependent in muscle and brain (Figure 4) and are sensitive to known TH agonists, for example, TRIAC (Figure 4). Interestingly, using the TH/bZIP construct we observed a lower threshold to TH agonists in the brain than in muscle. This sensitivity correlates with the main site of TH/bZIP expression, that is, the CNS. Indeed, this gene was first isolated from the diencephalon of *X. laevis* tadpoles (Denver et al. 1997), along with another 33 TH-regulated genes, including deiodinase and other metabolic enzymes. Some of these genes have also been isolated in neonatal mammals and chicks and could provide useful targets to be employed in screening approaches for thyroid disruption.

### The pretreatment protocol permits rapid detection and quantification of TH agonist action in germinally transgenic tadpoles.

Somatic gene transfer is ideal for comparing responses of different constructs and for setting up physiologic protocols. There is no need to establish founders or to maintain frog lines, and a number of test situations can be compared simultaneously. However, once a construct and a protocol have been selected, then germinal gene transfer becomes a much more efficient method for scaling up procedures for screening purposes. Using germinal transgenesis has the advantages of several hundred tadpoles per brood and a homogeneous population in terms of transgene insertion site and consequent regulatory controls. Furthermore, germinally transgenic tadpoles can also be used in a long-term assay, such as the XEMA test, to study impacts of longer term exposure to chemicals. The GFP signal can provide information on tissue-specific and developmental stage-specific actions during metamorphic progress, information that cannot easily be gleaned from the XEMA test on wild-type tadpoles (OECD 2004). Because TH/bZIP is expressed in all target tissues during amphibian metamorphosis and is highly responsive to TH in somatic gene transfer, we used it in germinal transgenesis. The genomic PCR fragment used with the luciferase reporter gene in somatic gene transfer experiments was fused to the eGFP reporter gene and inserted into an insulator-containing plasmid. The use of insulators helps overcome the influence of insertional position effects on transcriptional response, ensuring more homogeneous basal levels of expression of the transgene. Moreover, insulators have been shown to protect transgenes from methylation and maintain transgene expression in descendants (Kirillov et al. 1996) and have been successfully used in mice (Ciana et al. 2001). We applied the pretreatment protocol to TH/bZIP germinally transgenic tadpoles. TH/bZIP promoter-driven eGFP expression was significantly induced in brain and limb buds after a 2-day exposure to T_3_ (10^−8^ M) or to TRIAC (5 × 10^−8^ M), whereas no fluorescence was seen in caudal muscle during either natural metamorphosis or induced metamorphosis. This observation could be explained by the fact that we used only 400 bp of the TH/bZIP promoter, a fragment that might not include the enhancers responsible for targeting muscle expression.

### Applying the pretreatment protocol to germinally transgenic tadpoles allows rapid assessment of thyroid-potentiating effects of acetochlor.

Several current lines of research have revealed the widespread presence of hormonal pollutants in the environment. These disrupting substances of natural or synthetic origin interfere with hormone action affecting numerous functions, including homeostasis, reproduction, development, and behavior (Kavlock and Ankley 1996).

We tested the methodology described herein for assessing potential thyroid disruptors using the well-established thyroid disruptor acetochlor [2-chloro-*N*-(ethoxymethyl)-*N*-(2-ethyl-6-methylphenyl)acetamide]. This herbicide was introduced in 1994 in the midwestern United States (Kolpin et al. 1996). Acetochlor is persistent; surface water concentrations of acetochlor were found to be 0.2–4.5 nM at 1–3 months after application (Kolpin et al. 1996). Significant levels can still be detected in shallow groundwater 1 year after application (Crump et al. 2002). Acetochlor has been shown to alter thyroid axis functions in the rat (Wilson et al. 1996) and to alter the rate of metamorphosis in *Rana pipiens* as well as in *X. laevis* (Cheek et al. 1999; Crump et al. 2002). Using germinally transgenic tadpoles, we have shown that acetochlor amplifies the transcriptional response of the TH/bZIP promoter-driven eGFP reporter gene in the head region of pretreated tadpoles. This effect was observed with a weak, physiologic concentration of T_3_ (10^−10^ M), underlining the sensitivity of this *in vivo* method to assess actions of chemicals interfering with low physiologic amounts of T_3_. We also used somatic gene transfer to test the effects of acetochlor alone. However, we saw no effects on transcriptional response from TH/bZIP-luc (data not shown). Moreover, other authors using *Xenopus* (Crump et al. 2002) also saw no effect of 10^−8^ M acetochlor in the absence of T_3_ on endogenous TR-β and TH/bZIP expression in the tail. Similarly, using a *Rana* model, Cheek et al. (1999) showed that 7 days of exposure to acetochlor alone does not accelerate metamorphosis, even after a pretreatment with 10^−9^ M T_3_ during 3 days. These multiple observations indicate that acetochlor has no effect in the absence of T_3_. Moreover, concentrations of acetochlor in the environment are in the range of 10^−9^ M to 10^−8^ M, with the effect of acetochlor in presence of T_3_ occurring at 10^−8^ M (Cheek et al. 1999). Given that acetochlor persists in water, and that our results show that it can physiologically modify TH effects at environmentally relevant concentrations, our results bolster the concept that acetochlor contamination is a matter of acute environmental concern.

TH regulates a wide range of biologic processes during development and adult life. The fact that considerable numbers of compounds have the potential to interfere with different aspects of thyroid system function and TH action raises an urgent need for the development of an *in vivo* assay for detection of thyroid-axis–disrupting molecules. There is a long-standing debate in the field of endocrine disruption as to whether it is more important to reveal potential disrupting effects or to address the mechanisms of action underlying disruption. In the present study, we have opted to refine a test that will allow the detection of a wide range of disrupting chemicals rather than reveal mechanisms of action. Indeed, using the transcriptional response to natural ligand as an end point in an *in vivo* (vs. *in vitro*) context allows one to encompass a large range of potential interferences. For instance, if a chemical interferes with TH degradation, this should be picked up by a modification of the response to exogenous ligand. For example, the use of sodium perchlorate efficiently blocks metamorphosis by interfering with TH production. We have performed RT-PCR on such perchlorate-treated tadpoles and found decreased expression of TR-β (data not shown). This would be one example of a reduction of TH availability that is also played out at the level of a *TRE*-containing gene (the promoter of *TR*-β gene contains several functional TREs; Urnov and Wolffe 2001). Similarly, if a potential disruptor modulates receptor or comodulator availability, this, too, will be detected. Moreover, even if endogenous levels of TH are low in tadpoles at the stage used in these experiments, significant effects of disruptors on secretion and distribution of endogenous hormone will be detected. Thus, although the assay described does not address the eventual mechanisms of disruption, it will allow a broad spectrum of effects to be discerned.

A final point is that our model is both adaptable and flexible. The possibility exists of following two different hormone response systems simultaneously in the same tadpoles. This can be achieved by either using two separate plasmid constructs for transgenesis or using bicistronic plasmids (Fu et al. 2002). Furthermore, transgenesis in *X. laevis* takes advantage of the fact that the amphibian endocrine system has high similarity to that of other vertebrates and therefore offers the possibility of generalizing the approach to screen for other hormonal pollutants. This test is thus predictive for eventual hazards to both wildlife and human health.

In conclusion, we have developed a sensitive and rapid *in vivo* method to assess thyroid agonist activity, an approach that can potentially be combined with and applied to other hormonal axes.
